# Duration of androgen deprivation therapy with maximum androgen blockade for localized prostate cancer

**DOI:** 10.1186/1471-2490-11-7

**Published:** 2011-05-14

**Authors:** Naohiro Fujimoto, Tatsuhiko Kubo, Hideo Shinsaka, Masahiro Matsumoto, Shohei Shimajiri, Tetsuro Matsumoto

**Affiliations:** 1 Department of Urology, School of Medicine, University of Occupational and Environmental Health, Kitakyushu, 807-8555, Japan; 2 Department of Public Health, School of Medicine, University of Occupational and Environmental Health, Kitakyushu, 807-8555, Japan; 3 Department of Pathology and Cell Biology, School of Medicine, University of Occupational and Environmental Health, Kitakyushu, 807-8555, Japan

## Abstract

**Background:**

Primary androgen deprivation therapy (ADT) is a treatment option not only for advanced but also for localized prostate cancer. However, the appropriate duration for primary ADT for localized prostate cancer has not been defined and few studies have addressed this issue. In this study, we aimed to determine the appropriate duration of ADT for localized prostate cancer.

**Methods:**

Sixty-eight consecutive patients with localized prostate cancer who underwent a prostatectomy following neoadjuvant ADT were retrospectively reviewed. Factors associated with pT0, which is regarded as serious cancer cell damage or elimination, were investigated.

**Results:**

Of the 68 males, 24 (35.3%) were classified as pT0. The median duration of neoadjuvant ADT in the pT0 and non-pT0 groups was 9 months and 7.5 months, respectively (p = 0.022). The duration of neoadjuvant ADT from when PSA reached < 0.2 ng/ml to surgery was longer in the pT0 group than that in the non-pT0 group (median 5 months against 3 months, p = 0.011). pT0 was achieved in 5 of 6 patients (83.3%) who received ADT for ≥10 months after PSA reached < 0.2 ng/ml. No other clinical characteristics predicted conversion to pT0.

**Conclusions:**

Continuous ADT for ≥10 months after PSA reached < 0.2 ng/ml induced serious prostate cancer cell damage in most patients (> 80%) and may be sufficient to treat localized prostate cancer.

## Background

The advantages of primary androgen deprivation therapy (ADT) for localized prostate cancer continue to be controversial. A population-based epidemiological study indicated that primary ADT does not improve survival in the majority of patients with localized prostate cancer [1,2]. In contrast, Akaza et el. [3] demonstrated that primary ADT has favourable results for localized or locally advanced prostate cancer in Japanese males. They showed that the overall survival rate of Japanese males with prostate cancer was not different from that of normal Japanese males in the same age group. Although no evidence of benefit or sanction by guidelines has been reported, a substantial number of patients with localized prostate cancer receive primary ADT. In the United States of America, rates of primary ADT use in patients with localized prostate cancer have sharply increased, and primary ADT has become the second most common treatment approach after surgery for localized prostate cancer [4]. In Japan, primary ADT was administered in 39.5% patients with stage T1-T2 prostate cancer diagnosed in 2000 [5] and was the most common treatment in males with localized prostate cancer. Thus, primary ADT is a treatment option for advanced as well as localized prostate cancer. However, an appropriate duration of primary ADT for localized prostate cancer has not been defined. ADT is usually continued until disease progression or the occurrence of unacceptable adverse events. Is long-term primary ADT, ≥10 years in certain cases, required for treatment of localized prostate cancer? Long-term ADT is associated with adverse events such as bone fractures, diabetes, coronary heart disease and myocardial infarction [6,7]. Thus, the optimal duration for primary ADT should be defined that not only yields long-term cancer control or a cure but also reduces adverse events and costs and maintains quality of life. However, few studies have addressed this issue; therefore, it remains inconclusive. The appropriate duration of ADT can be defined by investigating the ADT duration required to kill most cancer cells. A useful strategy to understand the effect of ADT on cancer cells is a pathological evaluation of cancer status in prostatectomy specimens following neoadjuvant ADT. We explored the appropriate duration of primary ADT for localized prostate cancer by pathologically reviewing cancer status in radical prostatectomy specimens following neoadjuvant ADT with maximum androgen blockade (MAB).

## Results

Of the 68 patients, 24 (35.3%) were classified as pT0. The clinical characteristics, duration of neoadjuvant ADT and PSA levels of the pT0 and non-pT0 groups are listed in Table 1.

**Table 1:** Pre-treatment clinical characteristics and duration of ADT in the pT0 and non-pT0 groups

	pT0 (N = 24)	non-pT0 (N = 44)	p value
Age, yr			
Median	67	69	0.25
Range	56-76	54-75	
PSA level, ng/ml			
Median	10	11.3	0.261
Range	4.8-29.0	4.9-73.9	
Clinical stage (%)			
T1c	8 (33.3)	16 (36.4)	0.865
T2a	10 (41.7)	18 (40.9)	
T2b	3 (12.5)	7 (15.9)	
T2c	3 (12.5)	3 (6.8)	
Gleason score (%)			
≤ 6	7 (29.2)	12 (27.3)	0.227
7	11 (70.8)	27 (61.4)	
≥ 8	0 (0.0)	5 (11.4)	
Risk* (%)			
Low	4 (16.7)	9 (20.5)	0.887
Intermediate	10 (41.7)	16 (36.4)	
High	10 (41.7)	19 (43.2)	
Duration of ADT, mo			
Median	9	7.5	0.022
Range	3-19	3-29	
n PSA before PRx ** (%)			
< 0.2 ng/ml	24 (100)	39 (88.6)	0.219
> 0.2 ng/ml	0	5 (11.4)	

* According to D'Amico et al [18]

** nadir PSA before prostatectomy

**Figure 1:**
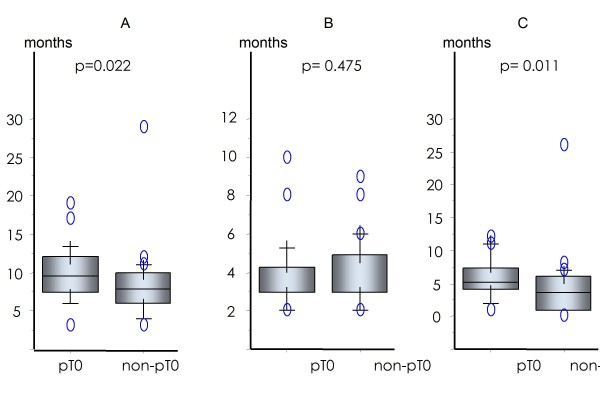
**Duration of ADT: total (A), before (B), and after (C) PSA reached < 0.2 ng/ml or its nadir**.

**Figure 2:**
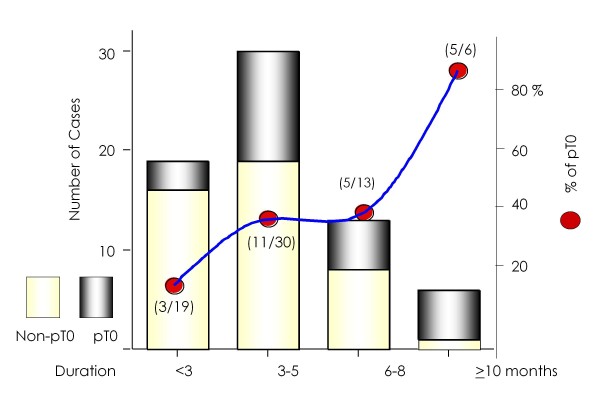
**Number and frequency of pT0 according to duration of ADT after PSA reached < 0.2 ng/ml or its nadir**.

The PSA values before and after ADT, clinical stages, Gleason score and risk classification did not predict conversion into pT0. The median duration of neoadjuvant ADT in the pT0 and non-pT0 groups was 9 months and 7.5 months, respectively (p = 0.022). When the duration of neoadjuvant ADT was divided into before and after PSA reached < 0.2 ng/ml or its nadir, the median duration before PSA reached < 0.2 ng/ml or its nadir was 3 months in both the pT0 and non-pT0 groups. In contrast, the median duration after PSA reached < 0.2 ng/ml or its nadir was 5 months and 3 months in the pT0 and non-pT0 groups, respectively. A longer duration after PSA reached < 0.2 ng/ml or its lowest value was significantly associated with pT0 classification (p = 0.011, Figure 1). pT0 frequency increased with a longer duration of ADT after the PSA reached < 0.2 ng/ml or lits nadir. Five of six (83.3%) patients who received ADT for ≥10 months after PSA reached < 0.2 ng/ml achieved pT0 (Figure 2) and this ratio was significantly higher than that in patients treated with ADT for shorter durations (p = 0.0099).

**Figure 3:**
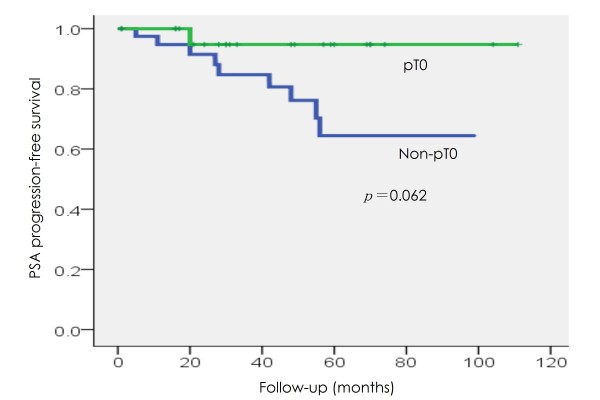
**PSA progression-free survival in the pT0 and non-pT0 groups**.

With a median postoperative follow-up of 31 months (range, 4-114 months), PSA progression was observed in one (4.2%) and nine (20.5%) patients in the pT0 and non-pT0 groups, respectively. Patients in the pT0 group had a tendency for longer PSA progression-free survival, although the difference was not statistically significant (p = 0.062) (Figure 3). Of all patients, only one in the non-pT0 group clinically progressed and died of prostate cancer 55 months following surgery.

## Discussion

Although the small number of positive biopsies, low Gleason scores (< 7) and low stages (cT1-2) may be associated with pT0 (8), only ADT duration was indicative of conversion into pT0 in the present study. The duration of ADT, particularly after PSA reached < 0.2 ng/ml, was significantly associated with pT0. Of the six patients who received ≥10 months of ADT after PSA reached < 0.2 ng/ml, five (83.3%) were classified as pT0. Whether viable cancer cells persist cannot be easily determined even by using immunohistochemistry or thin sections of specimens. Thus, pT0 may not be interpreted as the complete elimination of cancer. Kollermann et al. [8] did not observe a significant difference in PSA progression-free survival between patients classified as pT0 and non-pT0. However, we observed PSA progression in only one of 23 pT0 patients (4.3%) with a median follow-up of 35 months, and patients in the pT0 group had a tendency for longer PSA progression-free survival. Kitagawa et al. [9] showed that PSA progression was not observed in patients with a grade 3 pathological effect, regarded as pT0, with a median follow-up of 34.2 months. These observations indicate that pT0 can be regarded as an evidence of considerable cancer cell damage. Even when a small number of viable cancer cells remain, those cells will not develop clinically significant cancer in all cases. Thus, durations of ADT that induce pT0 could cause serious damage to cancer cells and achieve long-term control of localized prostate cancer.

Accumulated data illustrated the effect of ADT in combination with radiation therapy or prostatectomy. RTOG 92-02 [10] and EORTC 22961 [11] demonstrated the survival advantage of long-term ADT (24-36 months) compared with short-term ADT (4-6 months) in combination with radiation therapy. Because local treatment was identical in both groups, the systemic effect of 2-3 years of ADT could reduce or eliminate micrometastases, as well as any residual primary cancer cells, resulting in a better outcome. Most studies on neoadjuvant ADT before prostatectomy did not show a survival advantage [12], suggesting that neoadjuvant ADT does not completely eliminate cancer cells. In those studies, however, the duration of neoadjuvant ADT was 3-8 months. In intermittent androgen suppression (IAS), usually performed for localized, metastatic, or recurrent prostate cancer, the median on-therapy time is 3-9 months and PSA increases after 3-16 months of off-therapy [13]. These observations suggest that duration of ≤ 9 months may be insufficient, but 2-3 years of ADT may considerably eliminate cancer. Although whether 2-3 years of ADT is optimal remains unclear, the efficacy of limited-time, not lifelong, ADT may have the potential to obtain long-term control or cure for prostate cancer.

Labrie et al. [14] demonstrated the possibility of a cure with MAB of limited duration for localized prostate cancer. With a median follow-up of 4.9 years (for stage T2) and 5.6 years (for stage T3), non-PSA failure rates were 36%, 87.5% and 91.7% for patients previously treated with MAB for 3.5-6.5 years, 6.5-10 years and 10-11.7 years, respectively. No biochemical or clinical progression occurred in patients with localized cancer (T2) treated with MAB for ≥6.5 years. These results indicate that long term (> 6.5 years) continuous MAB offers the possibility of long-term control or a possible cure for localized prostate cancer. Although long-term (over 6.5 years) ADT may cure localized prostate cancer, whether such long-term ADT is really required remains unclear. In the study by Labrie et al., PSA failure was defined as an elevation of PSA > 1.0 ng/ml, which is too low to define cancer recurrence, because benign prostate cells can also produce PSA. Following radiation therapy with or without neoadjuvant ADT, PSA recurrence was defined as a PSA value of ≥2.0 ng/ml above the lowest value [15]. Then, duration ≤ 6.5 years of ADT may be sufficient to control localized cancer.

Cessation of ADT may not completely cure localized prostate cancer that may be obtained by long-term ADT, because pT0 does not always indicate complete elimination of cancer cells. Prostate cancer may recur following cessation of ADT in some patients. When PSA increased after cessation of ADT, Labrie et al. [14] observed a decrease of PSA in all cases by re-introduction of ADT. Re-introducing ADT can decrease the PSA level in most patients treated with IAS, which is at least as effective as continuous ADT [13]. These observations indicate that a vast majority of cancer cells remain androgen-sensitive after approximately 2 years of ADT. Rapid progression is unlikely, even in patients whose PSA levels rise after cessation of ADT, and patients could be treated successfully by re-introducing ADT at the time of recurrence. Therefore, cessation of ADT may not result in a poorer outcome compared with long-term continuous ADT in patients with localized prostate cancer.

The present study had some limitations. First, as all patients received MAB, the duration of ADT with mono-therapy, either castration or solely anti-androgen, is yet to be determined. Second, all patients in this study were Japanese. The response to ADT may differ among races, and Japanese males respond better to ADT than Caucasian males [16]. The appropriate duration may differ among races and should be defined by an investigation in other races. Third, the prevalence of pT0 was higher than that in previous reports [8,9,17]. We performed pathological evaluation using only hematoxylin and eosin (H & E) staining; the prevalence of pT0 may decrease if immunohistochemistry was employed. Relatively long durations of neoadjuvant ADT (58.8% were ≥8 months) and the race (Japanese) in our patients may also have been associated with the higher prevalence of pT0.

## Conclusions

Our results indicate that continuous ADT with MAB for 10 months after PSA reached < 0.2 ng/ml induced a marked therapeutic effect on cancer cells and may be a therapeutic option for patients with localized prostate cancer.

## Methods

The ethics committee of the University of Occupational and Environmental Health approved the study. Sixty-eight consecutive patients with clinically localized prostate cancer, who underwent a retropubic radical prostatectomy following neoadjuvant ADT between March 2001 and June 2010 at our institute, were reviewed retrospectively. Clinical staging was determined by a digital rectal examination, trans-rectal ultrasound, computed tomography scan and a bone scan. Pre-treatment risk categories were classified according to D'Amico et al. [18]. For neoadjuvant ADT, MAB with LH-RH agonist (leuproreline acetate or goserelin) and an anti-androgen (375 mg/day of flutamide or 80 mg/day of bicalutamide) was continued until prostatectomy. PSA level was assessed monthly until the prostatectomy.

Following surgery, no patient received adjuvant therapy and PSA was monitored every 3 months for 1 year, every 4 months for the next 2 years and every 6 months thereafter. PSA recurrence was defined as two consecutive values > 0.2 ng/ml. CTs and bone scans were performed when indicated.

Prostatectomy specimens were fixed in 10% buffered formalin and serially cut into 3-5-mm thick sections, representing transverse planes parallel to the initial apical and basal sections. The apical and basal transverse margins were sectioned perpendicularly to assess the prostatic apical and basal margins. Each prostate slide was processed into a whole mount section. One slide was routinely sectioned at 7 μm per block and stained with H & E. Specimens in which no cancer cells were detected or all cancer cells examined had non-viable features, such as nuclear pyknosis or karyolysis, were classified as pT0. Thus, pT0 suggested maximum damage to cancer cells. The pathological results of the prostatectomy specimens were grouped into pT0 and non-pT0.

Differences in pre-treatment clinical characteristics and in the duration of ADT between the pT0 and non-pT0 groups were analyzed with the Mann-Whitney *U*-test and the χ^2 ^test. An estimate of PSA progression following prostatectomy was calculated using the Kaplan-Meier method, and the significant difference between the pT0 and non-pT0 groups was calculated using the log-rank test; p < 0.05 was considered significant.

## Competing interests

The authors declare that they have no competing interests.

## Authors' contributions

NF and TM participated in the design of the study. TK carried out the statistical analysis. NF, HS, and MM collected patient data and follow-up information. SS performed pathological examination. NF drafted the manuscript. All authors read and approved the final manuscript.

## Pre-publication history

The pre-publication history for this paper can be accessed here:

http://www.biomedcentral.com/1471-2490/11/7/prepub
